# Diffusion Tensor Imaging Identifies Cervical Spondylosis, Myelitis, and Spinal Cord Tumors

**DOI:** 10.3390/diagnostics14121225

**Published:** 2024-06-11

**Authors:** Jiyuan Wang, Jing Huang, Bixiao Cui, Hongwei Yang, Defeng Tian, Jie Ma, Wanru Duan, Huiqing Dong, Zan Chen, Jie Lu

**Affiliations:** 1 Department of Radiology and Nuclear Medicine, Xuanwu Hospital, Capital Medical University, Beijing 100053, China; wjiyuan2023@163.com (J.W.); sainthj@126.com (J.H.); bixiao1311@163.com (B.C.); yhongw1993@163.com (H.Y.); tiandefeng0214@163.com (D.T.); spaceray@sina.com (J.M.); 2 Beijing Key Laboratory of Magnetic Resonance Imaging and Brain Informatics, Capital Medical University, Beijing 100053, China; 3 Department of Neurosurgery, Xuanwu Hospital, Capital Medical University, Beijing 100053, China; duanwanru@xwhosp.org (W.D.); chenzan66@163.com (Z.C.); 4 Department of Neurology, Xuanwu Hospital, Capital Medical University, Beijing 100053, China; shshtt@sina.com

**Keywords:** diffusion tensor imaging, fractional anisotropy, spinal cord tumor, cervical spondylosis, myelitis

## Abstract

Background: Diffusion tensor imaging (DTI) has been increasingly recognized for its capability to study microstructural changes in the neuropathology of brain diseases. However, the optimal DTI metric and its diagnostic utility for a variety of spinal cord diseases are still under investigation. Purpose: To evaluate the diagnostic efficacy of DTI metrics for differentiating between cervical spondylosis, myelitis, and spinal tumors. Methods: This retrospective study analyzed DTI scans from 68 patients (22 with cervical spondylosis, 23 with myelitis, and 23 with spinal tumors). DTI indicators, including fractional anisotropy (FA), mean diffusivity (MD), radial diffusivity (RD) and axial diffusivity (AD), were calculated. The Kruskal–Wallis test was used to compare these indicators, followed by Receiver Operating Characteristic (ROC) curve analysis, to evaluate the diagnostic efficacy of each indicator across disease pairs. Additionally, we explored the correlations of DTI indicators with specific clinical measurements. Results: FA values were significantly lower in tumor patients compared to those with cervical spondylosis (*p* < 0.0001) and myelitis (*p* < 0.05). Additionally, tumor patients exhibited significantly elevated MD and RD values relative to the spondylosis and myelitis groups. ROC curve analysis underscored FA’s superior discriminative performance, with an area under the curve (AUC) of 0.902 for differentiating tumors from cervical spondylosis, and an AUC of 0.748 for distinguishing cervical myelitis from spondylosis. Furthermore, a significant negative correlation was observed between FA values and Expanded Disability Status Scores (EDSSs) in myelitis patients (r = −0.62, *p* = 0.002), as well as between FA values and Ki-67 scores in tumor patients (r = −0.71, *p* = 0.0002). Conclusion: DTI indicators, especially FA, have the potential in distinguishing spondylosis, myelitis, and spinal cord tumors. The significant correlation between FA values and clinical indicators highlights the value of FA in the clinical assessment and prognosis of spinal diseases and may be applied in diagnostic protocols in the future.

## 1. Introduction

Spinal cord disorders are a significant cause of disability worldwide, affecting millions of individuals across various demographics [1,2]. Among these, cervical spondylosis, myelitis, and tumors have obvious clinical symptoms, which seriously affect the quality of life of patients. The economic and social burdens of these conditions are profound, with substantial costs associated with long-term care, rehabilitation, and lost productivity. Cervical spondylosis is particularly common, with studies suggesting that approximately 80 to 90% of individuals over the age of 50 are affected to some degree [3,4]. Myelitis, though less common, is a serious condition that can lead to weakness and sensory deficits [5]. Tumors of the spinal cord, while rare, are significant due to their potential malignancy and the complex challenges they pose in terms of treatment and management.

Cervical spondylosis, myelitis, and spinal tumors have overlapping symptoms of pain, stiffness, and neurological deficits; however, they are treated differently, and therefore an accurate diagnosis is urgently needed to improve patient outcomes. Although MRI (Magnetic Resonance Imaging) can provide important insights in clinical practice, its ability to distinguish these diseases is inadequate due to the lack of specificity of T2-weighted images. These images often show high signal characteristics across a range of pathological changes, including inflammation, edema, and tumors, leading to diagnostic challenges and poor clinical differentiation. This limitation is particularly problematic in conventional MRI, where the overlapping signals of various conditions blur the lines between structural degeneration, inflammatory processes, and neoplastic changes [6,7,8,9]. Conventional MRI cannot precisely distinguish between these diseases [10], so there is an urgent need for more specific imaging techniques to address the challenge of distinguishing between various spinal pathologies.

Diffusion tensor imaging (DTI) addresses the limitations of T2-weighted MRI in diagnosing spinal pathologies by offering detailed insights into white matter integrity and microstructure. DTI excels in differentiating pathological conditions through its ability to track water molecule movement, revealing microstructural changes [11,12]. DTI indicators, including fractional anisotropy (FA), mean diffusivity (MD), axial diffusivity (AD), and radial diffusivity (RD), are particularly sensitive in identifying spinal conditions, significantly improving diagnoses over T2-weighted MRI [12,13]. FA measures the directional dependence of water diffusion, indicating white matter integrity. MD represents the overall average rate of water diffusion, reflecting cellularity and tissue density. AD measures water diffusion along the main axis of fibers, associated with axonal integrity, while RD measures diffusion perpendicular to the fibers, often related to myelin integrity. Previous studies have shown that FA is more valuable than T2-weighted images in assessing spinal cord injury severity and its association with disability in multiple sclerosis (MS) patients [14,15]. This advanced imaging technique, by capturing the nuanced interactions between AD and RD, offers a nuanced approach to diagnosing spinal pathologies [16], far surpassing the capabilities of T2-weighted MRI.

Although several studies have demonstrated the unique advantages of DTI in the assessment of spinal cord diseases, such as demyelinating disease [17], inflammatory disease [18], and spinal cord injury [19], only a few have explored DTI’s differential diagnostic efficacy in spinal cord conditions. Raphael et al. examined the differential efficacy of DTI in sensory neuronopathy disorders and found that FA of the spinal cord was effective in differentiating between sensory neuronopathy patients and individuals with diabetic sensory–motor distal polyneuropathy [20]. Some researchers have also noted the efficacy of DTI in differentiating spinal cord tumors from tumor mimics [21,22]. However, comprehensive investigations into DTI’s differential diagnostic capabilities across various spinal cord lesions remain scarce.

Therefore, this study aims to harness the distinctive capabilities of DTI to advance the diagnostic precision and deepen our understanding of various spinal pathologies, namely, cervical spondylosis, myelitis, and spinal tumors. Firstly, we evaluate the discriminative power of DTI indicators to identify distinct patterns across these conditions. Secondly, we compare the diagnostic effectiveness of these indicators using ROC (Receiver Operating Characteristic) curves, which could evaluate the diagnostic performance by plotting the true positive rate against the false positive rate at various thresholds. Lastly, we correlate DTI indicators with clinical assessments, exploring their potential as biomarkers for disease severity and progression. Ultimately, by accomplishing these objectives, this study seeks to contribute a nuanced understanding and a more precise diagnostic approach to spinal cord pathologies.

## 2. Materials and Methods

### 2.1. Subjects

This is a retrospective study and ethical approval was waived by our Institutional Review Board. A total of 22 cervical spondylosis patients (13 females and 9 males), 23 cervical myelitis patients (13 females and 10 males), and 23 (9 females and 14 males) cervical tumor patients were recruited from the hospital outpatient service. We recruited spondylosis patients according to the criteria referred to in a study by Theodore et al. [23]. For the diagnostic criteria of myelitis, we refer to the discussion in this article [24]. The determination of a tumor patient is made by a combination of professional spinal neurosurgeons and radiologists for preliminary diagnosis, and the final diagnosis is based on medical record slices. There was no limit to disease duration. Sex and age were matched between the three groups.

### 2.2. Clinical Assessment

We collected clinical behavioral data to assist in assessing the severity of cervical myelitis and tumors. An Expanded Disability Status Score (EDSS) was used to assess the degree of neurological impairment in myelitis [25], which was recorded by an experienced neurologist (H.D., with more than 20 years of experience in neurology). For tumors, we evaluated tissue samples for the Ki-67 index, which is used to measure the proliferative activity of tumor cells [26].

### 2.3. MRI Acquisition

**Figure 1:**
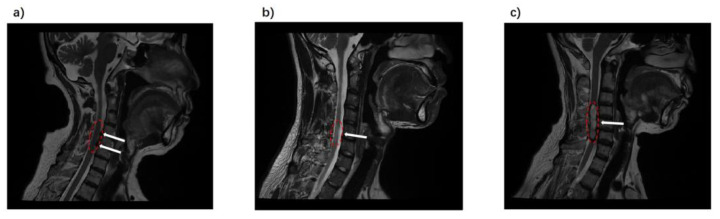
Examples of T2-weighted images of patients. (**a**) Cervical spondylosis. Disc herniation was seen at levels C4 to C6. (**b**) Cervical myelitis. High signal visible in C5/C6 segments. (**c**) Cervical tumors. The tumor lesion in the C4 to C6 segments was visible. Arrows and red border show the lesion location and segments.

Imaging was performed on a 3.0 T MR system (uPMR 790, United Imaging). DTI data were collected using an echo planar imaging (EPI) sequence, with 25 axial sections acquired (TR/TE = 5192/93 ms; flip angle = 90°; slice thickness = 5 mm; in-plane resolution = 0.9 × 0.9 mm^2^; and matrix size = 86 × 50; transversal acquisition). The diffusion gradients are a total of 32 different directions with b values of 0 and 600 s/mm^2^. High-resolution T2-weighted images were acquired with a MATRIX sequence (TR/TE = 1500/121 ms, matrix size = 256 × 256, slice thickness = 0.8 mm, voxel dimensions = 0.8 × 0.8 × 0.8 mm^3^, sagittal acquisition). To improve the imaging quality, several operations were carried out: The scan was positioned at the center of the C4/C5 intervertebral disc, and the scan frame covered from C2 to the lower edge of the C7 vertebrae. Diagnostic sequences commonly used in clinical practice were also scanned to identify specific lesion locations. Examples of diagnostic images for three different diseases are shown in Figure 1. It should be noted that DTI imaging was conducted as an additional scan following clinical diagnoses when ambiguities were present.

### 2.4. Data Processing

**Figure 2:**
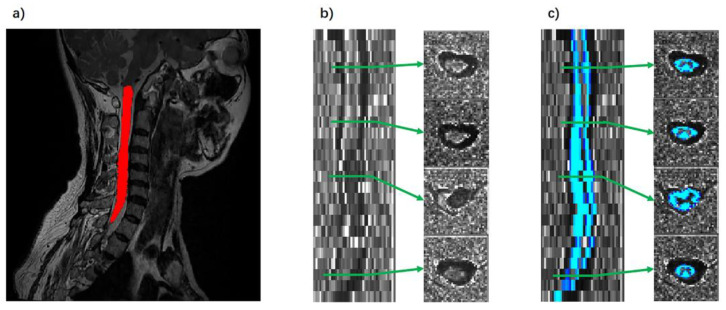
Demonstration of processing of structural and DTI images. (**a**) Automatic segmentation of structural images. (**b**) Fractional anisotropy (FA) maps of a patient with a cervical tumor. The tumor lesion showed abnormal signals on the spinal cord (third image from the top right). (**c**) Demonstration of white matter segmentation of FA maps. Red indicates the cord segmentation results. Blue indicates the white matter segmentation results. Green arrows indicate different slices.

MRI data were processed using the FMRIB Software Library version 6.0.3 (FSL; http://www.fmrib.ox.ac.uk/fsl/, accessed on 1 June 2023) and Spinal Cord Toolbox version 4.0.0 (SCT; https://spinalcordtoolbox.com/, accessed on 1 June 2023). Spinal cord segmentation was carried out with T2-weighted images using SCT (Figure 2a). Necessary manual adjustments were then made to ensure segmentation accuracy. Subsequently, each participant’s structural image was registered to the PAM50 template utilizing SCT registration tools (sct_register_to_template and sct_warp_template). This process involved creating both forward and backward warping fields. After the registration was determined, the PAM50 template was then inversely transformed into the native space of each image.

SCT is used to preprocess DTI data. For detailed steps, please refer to the study by De Leener et al. [27]. After the initial preprocessing, we executed the model fitting for the diffusion data. This process generated the FA and MD values. Subsequently, we calculated the AD and RD values. In the final step, we selected the spinal cord white matter template from PAM50′s standard space, specifically from segments C2 to C7, and registration was performed with each individual’s DTI images. The white matter segmentation and registration of the FA map are shown in Figure 2b,c. To extract the DTI indicators of the affected area, the ROI of the affected area was localized to specific cervical cord segments for each patient. Specific examples of affected areas are shown in Figure 1.

### 2.5. Statistics Analysis

Statistical analyses were performed by using SPSS software (version 23.0, IBM, Armonk, NY, USA). Difference tests were performed using the Kruskal–Wallis test and post hoc comparisons were made using Dunn’s test. To compare the diagnostic effectiveness of different DTI indicators for the three diseases, we plotted the ROC curves. Subsequently, we performed a Pearson correlation analysis of patients’ DTI indicators with clinical assessments. The significance tests were run with a two-tailed test with a significance level of 0.05.

## 3. Results

### 3.1. Patient Characteristics

There was no significant difference between the three groups of patients in terms of gender, age, height, and weight. The EDSS of myelitis patients was 1.63 ± 1.03 (mean ± standard deviation). The Ki-67 score of tumor patients was 15.30 ± 9.30 (%). Please refer to Table 1 for detailed data.

### 3.2. DTI Indicators in Spondylosis, Myelitis, and Spinal Tumors

**Figure 3:**
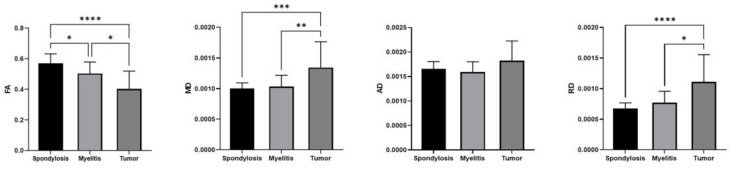
Comparisons of DTI indicators in the affected areas of the three groups. The FA values of tumor lesions were significantly lower than those of cervical spondylosis and myelitis lesions. Furthermore, the mean diffusivity (MD) and radial diffusivity (RD) values of the tumor patients were significantly higher than the other two groups. Axial diffusivity (AD) showed no significant difference. * *p* < 0.05, ** *p* < 0.01, *** *p* < 0.001, **** *p* < 0.0001.

These three diseases show different DTI patterns (Figure 3), with significant differences in FA values across the three groups (χ^2^(2) = 24.97, *p* < 0.0001). Post hoc comparisons revealed that FA values at the lesion site in tumor patients were significantly lower than those in the myelitis group (Z = 2.51, *p* = 0.04) and the cervical spondylosis group (Z = 5.00, *p* < 0.0001). Additionally, FA values in myelitis patients were significantly lower than those in cervical spondylosis patients (Z = 2.45, *p* = 0.04). The groups also differed significantly in MD values (χ^2^(2) = 17.10, *p* = 0.002). Tumor patients showed significantly higher MD values at the lesion site compared to both the myelitis (Z = 3.18, *p* = 0.0045) and cervical spondylosis groups (Z = 3.86, *p* = 0.0003). No significant difference in MD values was noted between myelitis and cervical spondylosis patients. Furthermore, significant differences were found in RD values among the groups (χ^2^(2) = 21.74, *p* < 0.0001). Tumor patients had significantly higher RD values at the lesion site compared to both the myelitis (Z = 2.84, *p* = 0.01) and cervical spondylosis groups (Z = 4.61, *p* < 0.0001). No significant difference in RD values was observed between myelitis and cervical spondylosis patients. Also, no significant differences were observed in AD values among the three groups (χ^2^(2) = 4.69, *p* = 0.10).

### 3.3. Discriminative Power of DTI Indicators in Spinal Pathologies

**Figure 4:**
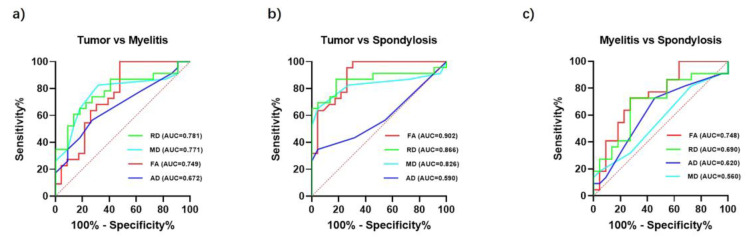
Diagnostic effectiveness of DTI indicators. (**a**) Effectiveness of differentiation between tumor and myelitis. (**b**) Effectiveness of differentiation between tumor and spondylosis. (**c**) Effectiveness of differentiation between myelitis and spondylosis.

ROC curves were utilized to assess the ability of four DTI indicators to distinguish between three spinal diseases: tumors versus myelitis, tumors versus cervical spondylosis, and myelitis versus cervical spondylosis (Figure 4). In contrasting tumors with myelitis, RD was found to be the most effective indicator, with an area under the curve (AUC) value slightly higher than that of FA (Figure 4a). The AUC values for these indicators were RD (0.781), MD (0.771), FA (0.749), and AD (0.672). ROC analysis indicated that FA was the most effective DTI indicator in distinguishing between spinal tumors and cervical spondylosis (Figure 4b). The AUC values for the indicators were as follows: FA (0.902), RD (0.866), MD (0.826), and AD (0.590). Similarly, FA emerged as the most discriminative DTI indicator in differentiating myelitis from cervical spondylosis (Figure 4c). The AUC values were ordered as FA (0.748), RD (0.690), AD (0.620), and MD (0.560). Overall, FA was effective in differentiating between all three diseases.

### 3.4. FA Correlations with Disease Severity in Myelitis and Spinal Tumors

**Figure 5:**
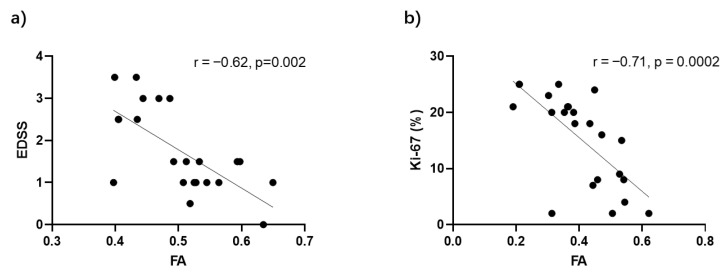
Correlation of FA values with patients’ clinical assessments. (**a**) FA values were significantly negatively correlated with Expanded Disability Status Scores (EDSSs) in patients with myelitis. (**b**) FA values were significantly negatively correlated with Ki-67 scores of tumor patients.

Since FA effectively differentiates between spinal tumors, myelitis, and cervical spondylosis, further analyses were conducted to explore its relationship with clinical assessments (Figure 5). In myelitis patients, a significant negative correlation was found between FA values at the lesion site and the EDSSs (r = −0.62, *p* = 0.002, Figure 5a). Meanwhile, in tumor patients, FA values showed a significant negative correlation with Ki-67 scores, a marker for cellular proliferation (r = −0.71, *p* = 0.002, Figure 5b).

## 4. Discussion

In the present study, DTI was employed to differentiate among spondylosis, myelitis, and spinal tumors, uncovering unique signatures for each pathology. Significant differences were observed in the values of FA, MD, and RD across these conditions. Furthermore, FA’s exceptional discriminatory power highlights its promising role in the differential diagnosis of spinal disorders.

The detailed analysis of DTI indicators in our study unveils complex microstructural alterations within the cervical cord affected by cervical spondylosis, myelitis, and tumors. Notably, the observed fluctuations in FA values may serve as indicators of changes in white matter integrity [28]. For spinal tumors, the marked reduction in FA values likely reflects the disturbance of normal white matter pathways that may occur as a consequence of tumor growth [29]. Such insights are invaluable, providing a deeper understanding of the tumor’s influence on the spinal cord’s neural architecture. On the other hand, the diminished FA values seen in myelitis can be interpreted as the result of inflammation-induced changes, such as edema and demyelination. These changes disrupt the uniform movement of water molecules along white matter tracts [30]. In comparison, patients with cervical spondylosis had the highest FA values. This suggests that compression did not significantly affect the microstructure of the spinal cord, and therefore, the integrity of the white matter was best preserved.

Additionally, the MD and RD values in our study exhibited distinct patterns that further assist in differentiating these conditions. Elevated MD values in tumor patients are generally considered indicative of increased extracellular space due to tissue destruction, a hallmark of tumor growth [31,32]. In contrast, the absence of significant differences in MD values between myelitis and cervical spondylosis patients suggests that the changes in white matter composition may not reach the level of pronounced disruption observed in tumors. The same holds for the result of the RD comparisons. Tumor cells can cause damage to the surrounding white matter myelin [33]. In addition to this, they may also affect the structure and arrangement of axons, resulting in a decrease in axonal density. All of these can cause an increase in RD values. In contrast, axons in patients with myelitis and spondylosis are retained more normally.

The high AUC values of FA in the ROC curve analysis imply that FA may be a reliable marker to identify spinal cord disease. The ROC curve is frequently utilized to evaluate the performance of diagnostic tests, where a higher AUC signifies superior discriminative capability. While FA values show variation between tumors and myelitis, they do not have the highest discriminative power. The best differentiation is offered by RD, characterized by its ability to reflect perpendicular water molecule diffusion, suggesting that changes in the myelin sheath or axonal integrity may be more pronounced in tumors than myelitis [34]. Despite the close AUC values between RD, MD, and FA, indicating a generally robust diagnostic utility across these DTI metrics, the slight edge of RD underscores the importance of considering the unique contributions of each DTI parameter in clinical assessments. A study by Hohenhaus et al. highlighted the utility of FA and MD in differentiating spinal tumors from inflammation [32], whereas our results suggest that RD has a slightly more significant diagnostic value in the context of tumors versus myelitis. Such discrepancies underscore the complex and multifaceted nature of spinal cord pathologies and the influence of underlying biological variations on DTI measurements.

In this study, the FA values of spinal white matter effectively distinguished between tumors and cervical spondylosis, achieving an AUC of 0.902. Concurrently, RD and MD also demonstrated commendable diagnostic discrimination. This aligns with the significant differences observed in FA, RD, and MD values among patients with cervical spondylosis and tumors. The cervical spinal cord of patients with cervical spondylosis included in this study showed only mild or no compression, leading us to postulate that their DTI indicators are closer to those of healthy individuals. Consistent with our findings, FA measurements have been proven to possess high sensitivity and specificity in detecting spinal pathologies in patients with extramedullary spinal canal tumors [35]. 

Similarly, FA demonstrated superior performance in distinguishing between cervical spondylosis and myelitis, suggesting that spinal inflammation primarily affects the anisotropic diffusion of water molecules. This aligns with previous studies showing significant FA reductions in the cervical spinal cord’s lateral and posterior segments in MS patients, closely linked to the severity of symptoms [36]. Furthermore, the identification of reduced FA values in areas that appear normal in conventional imaging confirms FA’s efficacy in detecting subclinical inflammatory changes [18]. Together, these insights highlight FA’s value as a sensitive biomarker for spinal cord inflammation, refining diagnosis for myelitis and improving treatment strategies.

The study’s findings emphasize the importance of FA as a non-invasive biomarker for assessing disease severity and predicting outcomes in myelitis and spinal tumors. The observed negative correlation between FA values and EDSSs in myelitis, as reported by Valsasina et al. for MS patients [14], highlights FA’s ability to reflect the severity of neurological impairments. This information can assist clinicians in developing targeted treatment plans. The correlation between FA values and Ki-67 scores in spinal tumors suggests that FA can be useful in identifying tumor activity and aggressiveness. This finding is supported by studies on brain tumors [37], indicating that DTI is a valuable tool for managing spinal conditions and gaining a deeper understanding of tumor biology in a non-invasive manner.

This study highlights the potential of DTI in distinguishing spinal pathologies. However, the generalizability of our findings may be affected by limitations such as a relatively small sample size and a cross-sectional design. Future research should focus on longitudinal studies with larger cohorts to validate and expand upon our results. Extending DTI’s application to a broader array of spinal conditions and enhancing imaging resolution can significantly improve diagnostic precision and deepen our understanding of spinal pathologies. It is important to note that our study did not differentiate between specific disease subtypes. Investigating the consistency and variability of DTI metrics across different spinal tumor types and inflammatory conditions is crucial. This will allow for the full utilization of DTI’s diagnostic and prognostic capabilities in both clinical practice and research.

## 5. Conclusions

DTI, especially FA, can distinguish between cervical spondylosis, myelitis, and spinal tumors. The strong correlation between FA values and clinical measurements highlights the important role of FA in evaluating and predicting spinal conditions, indicating its potential incorporation into future diagnostic protocols.

## Figures and Tables

**Table 1:** Summary of patient characteristics.

Characteristics	Spondylosis (*n* = 22)	Myelitis (*n* = 23)	Tumor (*n* = 23)
Gender (M/F) ^a^	9/13	10/13	9/14
Age (years) ^a^	53.68 ± 13.11	48.64 ± 17.48	46.74 ± 14.68
Height (cm) ^a^	165.18 ± 6.03	164.85 ± 9.70	158.83 ± 22.34
Weight (kg) ^a^	67.68 ± 11.65	66.77 ± 13.14	62.22 ± 18.67
EDSS	-	1.63 ± 1.03	-
Ki-67 (%)	-	-	15.30 ± 9.30

**^a^** There was no significant difference in sex, age, height, and weight between the three diagnosis groups. EDSS = Expanded Disability Status Score.

## Data Availability

Data are available upon request.
